# In situ experimental evidences for responses of abyssal benthic biota to shifts in phytodetritus compositions linked to global climate change

**DOI:** 10.1111/gcb.15882

**Published:** 2021-09-23

**Authors:** Hidetaka Nomaki, Eugenio Rastelli, Nanako O. Ogawa, Yohei Matsui, Masashi Tsuchiya, Elisabetta Manea, Cinzia Corinaldesi, Miho Hirai, Naohiko Ohkouchi, Roberto Danovaro, Takuro Nunoura, Teresa Amaro

**Affiliations:** ^1^ X‐star Japan Agency for Marine‐Earth Science and Technology (JAMSTEC) Yokosuka Japan; ^2^ Department of Marine Biotechnology Stazione Zoologica Anton Dohrn Fano Marine Centre Fano Italy; ^3^ Biogeochemistry Research Center (BGC) JAMSTEC Yokosuka Japan; ^4^ Research Institute for Global Change (RIGC) JAMSTEC Yokosuka Japan; ^5^ Institute of Marine Sciences National Research Council (ISMAR‐CNR) Venice Italy; ^6^ Department of Materials, Environmental Sciences and Urban Planning Polytechnic University of Marche Ancona Italy; ^7^ Department of Environmental and Life Sciences Polytechnic University of Marche Ancona Italy; ^8^ Stazione Zoologica Anton Dohrn Naples Italy; ^9^ Research Center for Bioscience and Nanoscience (CeBN) JAMSTEC Yokosuka Japan; ^10^ Department of Biology & CESAM University of Aveiro Aveiro Portugal; ^11^ Hellenic Center for Marine Research (HCMR) Heraklion Greece

**Keywords:** abyssal plain, benthic ecosystems, climate change, ecosystem functioning, isotope tracer, primary producers

## Abstract

Abyssal plains cover more than half of Earth's surface, and the main food source in these ecosystems is phytodetritus, mainly originating from primary producers in the euphotic zone of the ocean. Global climate change is influencing phytoplankton abundance, productivity, and distribution. Increasing importance of picoplankton over diatom as primary producers in surface oceans (especially projected for higher latitudes) is projected and hence altering the quantity of organic carbon supplied to the abyssal seafloor as phytodetritus, consequences of which remain largely unknown. Here, we investigated the in situ responses of abyssal biota from viruses to megafauna to different types of phytoplankton input (diatoms or cyanobacteria which were labeled with stable isotopes) at equatorial (oligotrophic) and temperate (eutrophic) benthic sites in the Pacific Ocean (1°N at 4277 m water depth and 39°N at 5260 m water depth, respectively). Our results show that meiofauna and macrofauna generally preferred diatoms as a food source and played a relatively larger role in the consumption of phytodetritus at higher latitudes (39°N). Contrarily, prokaryotes and viruses showed similar or even stronger responses to cyanobacterial than to diatom supply. Moreover, the response of prokaryotes and viruses was very rapid (within 1–2 days) at both 1°N and 39°N, with quickest responses reported in the case of cyanobacterial supply at higher latitudes. Overall, our results suggest that benthic deep‐sea eukaryotes will be negatively affected by the predicted decrease in diatoms in surface oceans, especially at higher latitudes, where benthic prokaryotes and viruses will otherwise likely increase their quantitative role and organic carbon cycling rates. In turn, such changes can contribute to decrease carbon transfer from phytodetritus to higher trophic levels, with strong potential to affect oceanic food webs, their biodiversity and consequently carbon sequestration capacity at the global scale.

## Graphical abstract

Schematic view of the natural settings of both 39°N and 1°N sites at western Pacific abyssal plain, as well as a synoptic view of the main highlights from the in situ incubation experiments conducted in this study on the responses of benthic biota to different phytoplankton supplies linked to global climate change. Benthic deep‐sea eukaryotes will be negatively affected by the predicted decrease in diatoms in surface oceans, especially at higher latitudes, while prokaryotes and viruses will otherwise likely increase their quantitative role and organic carbon cycling rates.

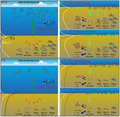

## 1 INTRODUCTION

The oceans buffer global climate change and its effects on terrestrial ecosystems by transporting carbon derived from the atmosphere to their deep‐sea interior and sediments (Reid et al., 2009). The deep sea is the largest, yet the least explored, environment on Earth. The seafloor deeper than 1000 m represents 63% of Earth's surface and it supports a high diversity of habitats and species (Grassle & Maciolek, 1992; Hessler & Sanders, 1967; Ramirez‐Llodra et al., 2010; Sogin et al., 2006). Furthermore, the deep sea is key in regenerating nutrients and sustaining global biogeochemical cycles, which in turn maintain primary and secondary production in the oceans (Danovaro et al., 2008; Thurber et al., 2014).

It has generally been assumed that deep‐sea habitats remain unchanged over long periods of time, as their remoteness was thought to buffer impacts from climate changes or anthropogenic activities. However, changes on a decadal to geological timescale linked to temperature and/or particulate organic carbon (POC) flux fluctuations have been reported in different deep‐sea areas and taxa (Ruhl et al., 2008; Ruhl & Smith, 2004; Smith et al., 2009; Yasuhara et al., 2008, 2009, 2014). The effects of human activities on deep‐water ecosystems are also increasingly been documented (Danovaro, Corinaldesi, Dell'Anno, & Snelgrove, 2017; Mora et al., 2013; Pham et al., 2014; Ramirez‐Llodra et al., 2011) and it is now acknowledged that deep‐sea ecosystems can respond dynamically to anthropogenic changes (Billett et al., 2001, 2010; Danovaro et al., 2010, 2020; Smith et al., 2009, 2020). These anthropogenic impacts on deep‐sea ecosystems are explained by the functional connectivity between the oceanic surface water and the deep seafloor via POC supply. Current scenarios of shifts in POC fluxes linked to global climate change predict increasing fluxes in the polar regions and decreasing fluxes in the temperate oceans, which cover most of the abyssal seafloor (Sweetman et al., 2017). Effects on benthic ecosystems have been shown to be proportional to the predicted changes in POC fluxes by presenting their relationships with the biomass of microbes, nematodes, macrofauna, megafauna, as well as sediment community oxygen consumption (Smith et al., 2008). However, there are still uncertainties that hamper reliable predictions of the impact of climate change on benthic deep‐sea ecosystems (Philippart et al., 2011). A major unanswered question is how the changes in food supply (i.e., changes in the composition and productivity of phytoplankton in the euphotic zone and subsequent sinking of organic matter [OM] through the water column) can alter deep‐sea benthic faunal activities (Dunlop et al., 2016). Moreover, most studies on the effects of POC fluxes on benthic organisms have been mainly focused on satellite‐based estimates compared with benthic census data, without direct *in situ* experimental evidence (Tittensor et al., 2011; Wei & Rowe, 2019). This is to date limiting our knowledge on the deep‐sea biota responses to the ongoing global climate change (Jöst et al., 2019; Yasuhara & Danovaro, 2016). Currently, the major primary producers at high latitudes (i.e., in typically eutrophic oceans) are generally diatoms, whereas those at low latitudes (i.e., in typically oligotrophic oceans) are generally picophytoplankton (including dinoflagellates and prokaryotes as well such as cyanobacteria; Uitz et al., 2006). Surface warming is expected to lead to more oligotrophic conditions, due to increased stratification (Smith et al., 2008; Sweetman et al., 2017). If the current trend in global climate change continues, enhanced stratification at high latitudes will likely cause a shift of the main primary producers in the phytoplankton community from diatoms to picophytoplankton (Barcelos e Ramos et al., 2007; Bopp et al., 2005; Moran et al., 2010; Smith et al., 2008; Tréguer et al., 2018). Thus, due to the global climate changes, not only the quantity but also the quality of POC fluxes could change.

The quality or type of food resources is indeed a further factor able to modulate the responses of benthic communities (Mayor et al., 2012; Nomaki et al., 2006), suggesting that changes in the surface primary producers will influence the abyssal benthic biota in future. Furthermore, the effect on the deep‐sea organisms of such shifts in the composition of the primary producers from diatoms to picophytoplankton might differ between oligotrophic and eutrophic systems, which currently display picophytoplankton versus larger eukaryotic phytoplankton (mostly diatoms), respectively, as main primary producers. The lack of information about the in situ responses of benthic abyssal ecosystems to such trophic changes currently limits our ability to predict how climate change will affect the overall ecosystem functions of the abyssal plain.

Here, to advance our knowledge on the impact of the projected shifts in the types of primary producers on deep‐sea benthic ecosystems, we conducted in situ feeding experiments using diatoms or cyanobacteria as analogues of different types of POC supply to the abyssal seafloor, by means of the human‐occupied vehicle (HOV) *Shinkai 6500*. Phytodetritus typically sinks from surface waters to deep‐sea ecosystems at 100–150 m day^−1^ reaching the abyssal seafloor within 1–2 months (Lampitt, 1985), and recent evidence suggests that such rates may be even faster (Agustì et al., 2015). In fact, intact and well‐preserved cyanobacterial and diatom cells have been reported to contribute to a substantial portion of the phytodetritus reaching depths >4000 m throughout the oceans (Agustì et al., 2015; Beaulieu & Smith, 1998; Lochte & Turley, 1988; Smith et al., 1996). Thus, in this study, we used frozen/thawed phytoplankton cells as analogues of phytodetritus, to provide novel insights into the use of diatom and cyanobacterial phytodetritus as a food source by abyssal organisms.

To compare the responses of abyssal benthic biota in eutrophic and oligotrophic oceanic areas, we selected two western Pacific abyssal plain experimental sites: one eutrophic station, 39°N (39°00.0ʹN 146°00.1ʹE, 5260 m water depth) and one oligotrophic station, 1°N (1°15.0ʹN, 163°14.8ʹE, 4277 m water depth). These stations exhibit substantial differences in POC fluxes to the seafloor and hence, benthic biogeochemistry and abundances of benthic organisms (Nomaki et al., 2021). At both sites, to compare the responses by different size classes of organisms, we analyzed the uptake of ^13^C and ^15^N by eukaryotes (including mega‐, macro‐, and meiofauna) as well as the responses of prokaryotes and viruses and overall mineralization of the labeled phytoplankton. Moreover, to further investigate if the different biotic components differed in the timing of their responses to phytoplankton supply, our experiments included different time point incubations (i.e., over 1–2 days and 2 months). Finally, to test if the more intense bioturbation of surface sediments at Station 39°N (Nomaki et al., 2021) could play a role in influencing the processing of phytoplankton in abyssal sediments, we completed our tests by analyzing the mixing and mineralization of the isotopically labeled phytoplankton down the sediment vertical profile.

## 2 MATERIALS AND METHODS

### 2.1 Study sites

The in situ experiments were carried out during September–November 2013 (JAMSTEC cruises YK13‐09 and YK13‐12 of R/V *Yokosuka*) at Station 1°N and May–July 2014 (YK14‐06 and YK14‐12) at Station 39°N for up to 58 days using the deep‐diving HOV *Shinkai 6500*. We selected abyssal plain sites without depressions or hills to minimize any topographic effects on faunal composition and abundance (Durden et al., 2015; Stefanoudis et al., 2016). POC fluxes to the seafloor were 485 mg C m^−2^ year^−1^ at Station 1°N in 2013 and 1086 mg C m^−2^ year^−1^ at Station 39°N in 2014 (Table 1), based on the satellite‐derived chlorophyll data and equation described by Lutz et al. (2007). At Station 1°N, the highest net primary production (NPP) was recorded in April (412 mg C m^−2^ day^−1^) and lowest in November (175 mg C m^−2^ day^−1^) during 2013. At Station 39°N, a larger spring bloom was recorded with a NPP peak of 2585 mg C m^−2^ day^−1^ and lowest NPP value in January (278 mg C m^−2^ day^−1^) during 2014, typical cases for high‐latitude ocean that exhibits strong seasonality in NPP. More information regarding the two abyssal sites selected for our in situ experiments can be found in the studies by Amaro et al. (2019) and Nomaki et al. (2021).

**TABLE 1:** Added algae and their C and N isotopic compositions and POC flux to the seafloor at stations 39°N and 1°N

Site	Algae	^15^N atom %	^13^C atom %	Added C amount for open‐box‐type (mg C m^−2^)	Annual POC flux at this site (mg C m^−2^)^a^
39°N	*Chaetoceros sociale*	11.7	15.6	215.0	1086
	*Synechococcus* sp.	16.9	11.4	295.5	
1°N	*C. sociale*	17.9	12.0	137.5	485
	*Synechococcus* sp.	17.4	7.9	254.2	

^a^ Calculated according to the study by Lutz et al. (2007).

### 2.2 Incubation of phytoplankton labeled with stable isotopes

For the in situ incubation experiments, we cultivated the diatom *Chaetoceros socialis* (common in eutrophic areas of the ocean) strain NIES 377 (National Institute for Environmental Studies, Japan) and the cyanobacterium *Synechococcus* sp. (widespread in oligotrophic areas of the ocean) strain NIES 969 (Table 1). These were isotopically labeled by additions of ^13^C‐labeled NaHCO_3_, ^15^N‐labeled NH_4_Cl, and ^15^N‐labeled NaNO_3_ to f/2 medium (diatom) or ESM medium (cyanobacterium), as previously described, with a few modifications (Nomaki et al., 2005, 2006). Cells at the early stationary phase were harvested by centrifugation at 800× g for 5 or 10 min. The cells were rinsed thrice with unlabeled f/2 or ESM medium and stored at −30°C before the in situ incubation experiments. The cells ranged from 7.9 to 15.6 for ^13^C atom% and from 11.7 to 17.9 for ^15^N atom% (Table 1).

### 2.3 In situ feeding experiments and sampling

At both stations, our in situ feeding experiments included two types of incubation systems: open‐box‐type and a mesh‐type incubation chambers (Table S1; Figures S1 and S2, respectively).

The open‐box‐type chambers were designed to investigate the responses of the benthic assemblages (meiofauna and macrofauna, prokaryotes, and viruses) to the isotopically labeled phytoplankton. Each of these chambers covered 625 cm^2^ (25 cm × 25 cm) of the seafloor and was equipped with six 50‐ml syringes containing ^13^C‐ and ^15^N‐labeled diatoms or cyanobacteria. At each site, four of these open‐box‐type chambers were deployed in situ on the seafloor using the manipulators of HOV *Shinkai 6500*. Then, the ^13^C‐ and ^15^N‐labeled diatoms or cyanobacteria contained in each set of syringes were injected onto the part of the seafloor covered by the respective chambers (Figure S1) to simulate different phytoplankton supplies and to follow the fate of these phytoplankton within the abyssal food web. At each site, two of the four open‐box‐type chambers were fed with diatoms, while the other two were fed with cyanobacteria, totaling eight open‐box‐type chambers used for this study (Table S1). Areal concentrations of the two isotopically labeled phytoplankton corresponded to 215 mg C m^–2^ at 39°N and 137.5 mg C m^–2^ at 1°N (diatoms) and 295.5 and 254.2 mg C m^–2^ (cyanobacteria), respectively (Table 1). These phytoplankton concentrations corresponded to roughly 20% (diatom)–27% (cyanobacteria) and 28% (diatom)–52% (cyanobacteria) of the annual POC flux at 39°N and 1°N, respectively. One day after the addition of phytoplankton, the lid was automatically opened by galvanic time‐release triggers (International Fishing Devices) to minimize any potential chamber/enclosure effects such as hypoxia in the chamber. Three sediment cores were collected from each chamber after 1 and 58 days (39°N) or 2 and 51 days (1°N), using independent H‐type push corers (each of inner diameter, 82 mm; length, 320 mm). One dedicated chamber from either diatom or cyanobacteria treatments was sampled at each incubation time point (Table S1).

The mesh‐type incubation chambers were designed to investigate the role of holothurians, the key megafauna at the investigated sites. These incubation chambers (1000‐cm^2^ inner surface area) were equipped with two 50‐ml syringes containing ^13^C‐ and ^15^N‐labeled diatoms or cyanobacteria. Six mesh‐type chambers were deployed on the seafloor with a single holothurian individual in each chamber at 39°N (*Scotoplanes globosa*) and three at 1°N (*Psychropotes longicauda* or *Deima validum*; Table S1). At 39°N, the syringes of all six chambers were filled with ^13^C‐ and ^15^N‐labeled cyanobacteria. After deployment on the seafloor, each holothurian was kept inside the chamber, and the ^13^C‐ and ^15^N‐labeled cyanobacteria were injected onto the seafloor inside the chamber using manipulators of HOV *Shinkai 6500*. At 39°N, three of the six chambers were retrieved 1 day after deployment, while the other three were retrieved after 58 days (Table S1). Each mesh‐type chamber was gently removed by a manipulator, and each holothurian was collected using a suction sampler connected to a rotary sample container with multiple chambers. The sediment where the chamber had been deployed was then collected using the push corer to collect meiofauna and macrofauna for isotopic analyses. At 1°N, ^13^C‐ and ^15^N‐labeled diatoms were added into one chamber, and ^13^C‐ and ^15^N‐labeled cyanobacteria were added into two chambers (Table S1). Two days after deployment, holothurians in one cyanobacteria chamber and the diatom chamber were sampled. The one in the other cyanobacteria chamber was sampled after 51 days. At both stations, all holothurians were still alive at the time of sampling.

All sediment samples were brought back in the casing with ambient water to minimize possible warming during transfer, and the sediment cores recovered onboard from both the open‐box‐type and mesh‐type chambers were kept in a cold room (4°C), before sample processing. The overlying water was gently sampled for analyses of the C isotopic composition of dissolved inorganic carbon (DIC) and nutrient concentrations (see Section 2.8). Sediment cores were sliced horizontally into sections corresponding to depths of 0–0.5, 0.5–1, 1–2, 2–3, 3–5, 5–7, 7–10, and 10–15 cm. At 1°N, sediments were not sampled below the depth of 5 cm. The sliced sediment samples collected from the open‐box‐type chamber were subdivided for the analyses of macrofauna and meiofauna (~25 ml per cm of sediment), viral abundance and production rates (2 ml), extracellular enzymatic activity (2.5 ml), prokaryotic cell numbers (1 ml), and analyses of OM and porewater (~22 ml), as described below. The sediment samples from mesh‐type chambers were used entirely for faunal analyses, as described in Section 2.6.

The samples obtained from each experiment are hereafter referred as Diatom‐1d, Diatom‐58d, Cyano‐1d, and Cyano‐58d at Station 39°N, and Diatom‐2d, Diatom‐51d, Cyano‐2d, and Cyano‐51d at Station 1°N. In addition to the samples from these experimental treatments, background sediment samples were also collected to quantify natural abundances and stable isotope signatures of the benthic organisms, as well as the sediment geochemical parameters (Nomaki et al., 2021). Background sediment samples for the analyses of prokaryotes, extracellular enzymatic activities, virus abundances and production rates were also collected at the start of the experiment and at each time point of the incubations during the experiments.

### 2.4 Analysis of prokaryotes, viruses, and enzymatic activities

Prokaryotic and viral abundances in the surface 0–1 cm sediment layer were determined by epifluorescence microscopy after extraction of cells and viruses from the sediment by sonication in 0.02 µm prefiltered pyrophosphate (final concentration, 5 mM) seawater solution and subsequent staining with SYBR Green I (Danovaro, 2009; Manea et al., 2019). Viral production rates and extracellular enzymatic activities (aminopeptidase and β‐glucosidase) in the surface 0–1 cm sediments were assessed on the research vessel by means of time course experiments in the dark and at in situ temperature, as described in the study by Manea et al. (2019). Briefly, viral production rates were assessed onboard the research vessel using the dilution‐based procedure (Dell'Anno et al., 2009; Rastelli et al., 2016) and samples from the top 1 cm of undisturbed deep‐sea sediments diluted immediately after retrieval with virus‐free seawater (prefiltered onto 0.02‐µm filters), collected at the sediment–water interface of each site (final dilution: 1:10 sediment: seawater vol:vol; Manea et al., 2019). Viral counts were conducted over time during the incubations, and viral production rates were determined from linear regression analyses of the increase in viral abundances over time (Danovaro et al., 2016; Dell'Anno et al., 2009; Rastelli et al., 2016). Enzymatic activities were determined for both aminopeptidase and β‐glucosidase as a proxy of the metabolic response of prokaryotes (either for protein or for carbohydrate enzymatic degradation) to the in situ addition of diatoms or cyanobacteria. Briefly, enzymatic activities were conducted by the analysis of the cleavage rates of the artificial fluorogenic substrates (L‐leucine 7‐amido‐4‐methylcoumarin hydrochloride and 4‐methylumbelliferyl‐b‐D‐glucopyranoside, respectively; Sigma Chemicals), under saturating substrate concentrations (Danovaro, 2009). Sediment subsamples were diluted with 0.02 µm prefiltered sterilized seawater and incubated with each fluorogenic substrate for 1–2 h. The fluorescence of the samples was measured fluorometrically immediately after the addition of the substrate and after the incubation, checking for linearity of the increase in fluorescence during the incubation period and converting into enzymatic activity based on standard curves obtained using the respective standards (Sigma Chemicals; Danovaro, 2009). Extracellular enzymatic activities of frozen and thawed isotope‐labeled diatoms and cyanobacteria used for the supply experiments were also evaluated in the same way and showed no detectable values (data not shown). All sediment samples were analyzed in three replicates. These were collected from three push corers collected either in one of the available in situ chambers at each sampling time during the incubation period (for the diatoms or cyanobacteria treatments), or in the background undisturbed sediment spots next to the chambers (for both the “initial” sediments and the “control” sediments collected at each sampling time during the incubation period). All data were normalized to sediment dry weight after desiccation (48 h at 60°C).

### 2.5 Macrofaunal and meiofaunal analyses

Macrofauna and meiofauna for isotopic composition measurements were isolated from the sediment according to the protocol described in the study by Danovaro (2009). In brief, all sliced sediment was fixed immediately in 10% buffered formalin diluted in seawater and mixed with Rose Bengal dye (0.05 g L^−1^). In the laboratory on land, each layer was carefully washed with filtered artificial seawater through 250‐μm and 32‐μm mesh sieves. Organisms retained on the 250‐μm sieve, hereafter referred as macrofauna, were sorted into major taxa and then identified to family or genus level, where possible. Each specimen was stored for the analyses of C and N isotopic compositions.

Particles remaining on the 32‐μm sieve were resuspended and centrifuged three times with colloidal silica (Ludox HS40; Sigma‐Aldrich) according to Danovaro (2009). The supernatants were transferred to a Petri dish with a 5‐mm grid on the bottom, and the Rose Bengal‐stained organisms, hereafter referred as meiofauna, were sorted to the higher taxon level and counted under a binocular stereoscopic microscope according to the study by Giere (2009).

### 2.6 Stable isotopic compositions of sediment and organisms

About 1 ml of each sediment sample was used for the analyses of total organic carbon (TOC), total nitrogen (TN), and their isotopic compositions (δ^13^C, δ^15^N; see details in Amaro et al., 2019; Nomaki et al., 2019). The samples were freeze‐dried, powdered, and weighed into precleaned silver capsules. The samples were decalcified with 2 M HCl and then dried on a hotplate at 60°C. Dried silver capsules containing decalcified samples were sealed into precleaned Sn capsules before isotopic analysis.

Macrofaunal and meiofaunal samples were put directly into precleaned tin capsules and dried at 60°C to remove water and to determine the dry weight. Typically, most macrofauna were measured with single individuals, whereas copepods (10–70 individuals per sample), nematodes (78–459 individuals per sample), foraminifera (12–90 individuals per sample), and other meiofauna individuals were pooled into one sample. They were then decalcified with 0.1 M HCl, completely dried again, and then sealed using precleaned forceps.

Carbon and N isotopic compositions, along with the TOC and TN content of decalcified samples, were determined by three different isotope ratio mass spectrometers (IRMS) coupled to elemental analyzer (EA) systems according to their sample types. In brief, sediment samples were analyzed by Flash EA1112‐ConFloIV DELTA V Advantage System (Thermo Fisher Scientific) at SI Science Co. ltd.; macrofauna samples were analyzed by Flash EA1112‐ ConFloIV DELTA plus Advantage System (Thermo Fisher Scientific) at JAMSTEC; and meiofauna were analyzed by a modified system of Flash EA1112‐ConFloIII Delta plus XP (Thermo Finnigan; Ogawa et al., 2010) at JAMSTEC. The isotope ratios were expressed in delta‐notation as δ*X* = [(*R*_sample_/*R*_standard_) – 1] × 1000, where *X* is ^13^C or ^15^N and *R* is the ratio ^13^C:^12^C or ^15^N:^14^N. Analytical errors for δ^13^C and δ^15^N standards were both within ±0.18‰ (SD, 1*σ*) for all three EA/IRMS.

Isotopic labeling in organisms due to their ingestion/assimilation of ^13^C‐ and ^15^N‐labeled diatoms or cyanobacteria was quantified as the difference in the δ^13^C or δ^15^N values between incubated samples and natural background samples (Nomaki et al., 2021) as ∆δ^13^C and ∆δ^15^N, respectively (Tables S2–S6). Due to the fact that natural background samples exhibit variations in their isotopic compositions (Nomaki et al., 2021), we subtracted 2*σ* (SD) of the natural background δ^13^C or δ^15^N of each taxon from the ∆δ^13^C and ∆δ^15^N values, respectively. We considered that samples for which the positive ∆δ^13^C − 2SD or ∆δ^15^N − 2SD values indicate the organisms had ingested or assimilated ^13^C‐labeled or ^15^N‐labeled phytoplankton. The background isotopic compositions of some macrofaunal taxa measured in experimental samples, namely Decapoda, Glyceridae, Sphaerodoridae, Syllidae, Pilargidae, Solenogastre, and Trichobranchidae, were not measured in natural samples (Tables S2; Nomaki et al., 2021). For these taxa, the highest natural δ^13^C or δ^15^N values of any macrofaunal taxon +2SD of the associated values at each site were used for the calculation (Tables S3 and S4). As both the δ^13^C and δ^15^N varied as a function of the feeding ecology and metabolic characteristics of the organisms, the use of the highest value +2SD of any organism in the community should underestimate ingestion by fauna. Uptake or assimilation reported in this study should therefore be considered to be conservative estimates.

### 2.7 Porewater nutrient concentrations and ^13^C‐DIC analyses

Porewater was extracted from natural background sediments and open‐box‐type experimental samples by centrifugation. From the same open‐box incubation chamber, one sediment core was used for the analysis of nutrient concentrations, and the other was used for the analysis of DIC δ^13^C values.

For nutrient analyses, ~20 ml of overlying water was gently collected using a tube. A sample of ~22 ml of sediment from each 1‐cm interval of the sediment core was transferred to a 50‐ml conical tube and centrifuged at 2600× g for 5 min. The overlying water and the extracted porewater were filtered through a 0.45‐µm membrane filter. The porewater samples were stored at −25°C before nutrient analyses back in land‐based laboratory. Nutrient concentrations were measured with a continuous‐flow analyzer (BL‐Tech QUAATRO 2‐HR system; Nomaki et al., 2016). The precision of the phosphate, nitrate, nitrite, and ammonium measurements, based on duplicate measurements, were ±0.17%, ±0.17%, ±0.16%, and ±0.38%, respectively.

For ^13^C‐DIC analyses, ~20 ml of overlying water was gently collected using a tube and directly transferred into a 20‐ml glass vial. Sediment porewater samples extracted by centrifuging were also transferred into a 20‐ml glass vial. Immediately after collection, the samples were fixed with HgCl_2_, sealed with a rubber septum–aluminum cap, and then stored at 4°C before further analyses (Nomaki et al., 2011). The δ^13^C of the DIC was measured with a stable isotope mass spectrometer (IsoPrime; GV Instruments), as described in the study by Nomaki et al. (2011).

### 2.8 Statistical analysis

To identify significant differences in prokaryotic abundance, enzymatic activities, viral abundances, and viral production rates in the experimental systems at Stations 39°N and 1°N, after checking for homogeneity of variance, analysis of variance was conducted on the Euclidean distance similarity matrices. Post hoc pairwise tests were carried out when significant (*p* < .05) differences were detected. For each site, the factors included in the analyses were *time* (initial, intermediate, and final incubation time) and *type* (background samples—either initial background samples or background controls collected at the different time points during the incubation period—diatom addition, and cyanobacteria addition; Tables S7 and S8). All tests were carried out in the software package Primer v6 + PERMANOVA (Anderson et al., 2008).

## 3 RESULTS

### 3.1 Mixing and mineralization of phytoplankton labeled with ^13^C and ^15^N

The Δδ^13^C and Δδ^15^N profiles of total OM in the sediments at Station 39°N showed high values down to 2 cm in Diatom‐1d and subsurface peaks at 4 cm in Cyano‐1d (Figure S3). The Δδ^15^N profile exhibited modest enrichment in the surface sediment (∆δ^15^N values of <5‰) in Diatom‐58d and Cyano‐58d and no Δδ^13^C values exceeded 1‰ in the same cores. At Station 1°N, ^13^C‐ and ^15^N‐ enrichments were observed at depths shallower than 2 cm in Diatom‐51d and Cyano‐51d.

**FIGURE 1:**
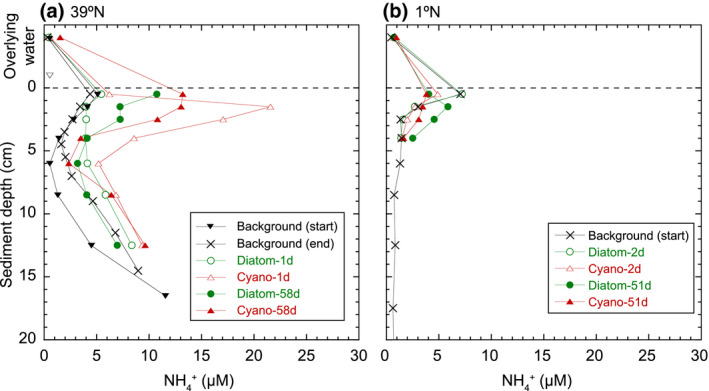
Profiles of ammonium concentrations in the porewater extracted from the cores of natural sediments and of sediments to which phytoplankton were added at Stations 39°N (a) and 1°N (b). Ammonium concentrations in the subsurface sediment increased at Station 39°N after the addition of phytoplankton probably due to their mineralization. Values above 0 cm indicate concentrations in the water overlying the sediments

Porewater ammonium concentrations in sediments at Station 39°N increased after the in situ experiments relative to background sediments, particularly between 2 and 5 cm (Figure 1a). At Station 1°N, in contrast, no clear increases were observed, although some variations were observed at 0–5 cm.

### 3.2 Faunal responses to phytoplankton labeled with ^13^C and ^15^N

At Station 39°N, two holothurian individuals (*S. globosa*) were slightly enriched in ^15^N (∆δ^15^N − 2SD of 0.7‰ and 2.0‰) in Cyano‐58d, but their δ^13^C values were within the range of natural variation (Table S2). Apparent enrichment in either ^13^C or ^15^N was not observed in the three specimens of *S. globosa* from Cyano‐1d.

At Station 1°N, a holothurian *P. longicauda* collected from Cyano‐2d was slightly enriched in ^13^C (∆δ^13^C − 2SD of 1.5‰), but there was no enrichment in ^15^N (Table S2). The other holothurian individuals were neither enriched in ^13^C nor in ^15^N in Diatom‐2d (*D. validum*) and Cyano‐51d (*P. longicauda*).

**FIGURE 2:**
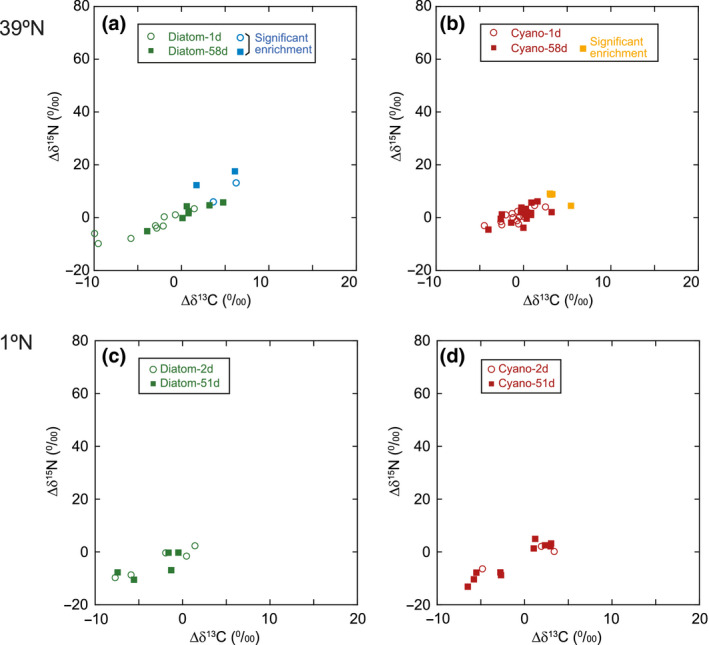
Enrichments in ^13^C and ^15^N in macrofauna after 1 and 58 days (39°N; a, b) or after 2 and 51 days (1°N: c, d) following phytoplankton additions. Limited numbers of macrofauna were significantly enriched in ^13^C or ^15^N by labeled cyanobacteria (orange) or labeled diatoms (blue), which has the ∆δ^13^C and ∆δ^15^N values greater than the average +2*σ* (SD) of natural background samples

Several species of macrofauna were enriched in ^13^C and ^15^N at 39°N, particularly at 58 days (Figure 2a,b; Table S3). A bivalve mollusk exhibited ∆δ^13^C − 2SD value of 1.5‰ and ∆δ^15^N − 2SD value of 7.4‰ in Diatom‐1d. A bivalve and an annelid (Paraonidae) were also enriched in ^15^N (∆δ^15^N − 2SD values of 6.5‰ and 11.8‰, respectively) in Diatom‐58d. Sabellid and spionid polychaetes (Annelida) as well as an unidentified macrofauna were slightly enriched in ^13^C or ^15^N (∆δ^15^N − 2SD of up to 3.2‰). No enrichments in ^13^C or ^15^N were detected in macrofauna specimens from Cyano‐1d, including annelids. At 1°N, no enrichments in ^13^C or ^15^N were observed in macrofauna (e.g., isopods, copepods, nematodes, annelids) incubated with labeled diatoms or cyanobacteria during any of the incubations (Figure 2c,d; Table S4).

**FIGURE 3:**
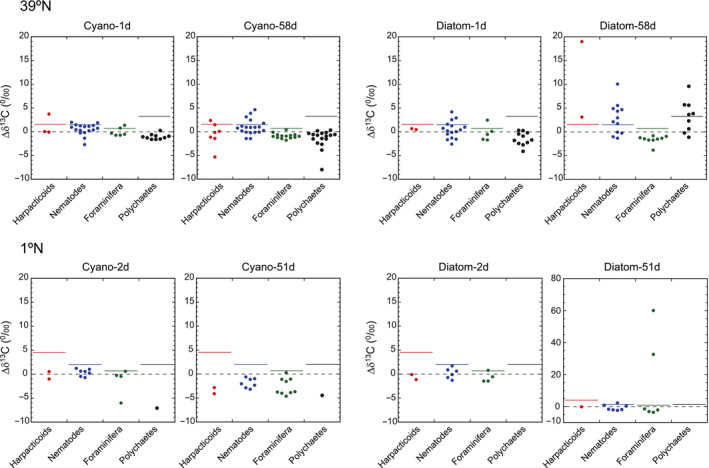
Enrichments in ^13^C in meiofauna 1 and 58 days (39°N) or 2 and 51 days (1°N) after additions of phytoplankton, showing significant uptake of isotope‐labeled phytoplankton particularly at Station 39°N. The colored bars indicate ∆δ^13^C values greater than the average +2*σ* (SD) of natural background samples

At Station 39°N, diverse meiofaunal taxa, except bivalves, were enriched in ^13^C and ^15^N, especially in Diatom‐58d and Cyano‐58d (Figure 3; Table S5). Meiofaunal‐size polychaetes were enriched in ^13^C and ^15^N in Diatom‐58d (∆δ^13^C − 2SD of up to 6.4‰ and ∆δ^15^N − 2SD of 27.2‰), but no analogous enrichment was observed in Cyano‐58d (i.e., there were no positive ∆δ^13^C − 2SD values, and ∆δ^15^N − 2SD values never exceeded 1.4‰). Harpacticoid copepods were clearly enriched in ^13^C and ^15^N in Diatom‐58d (∆δ^13^C − 2SD of up to 17.4‰ and ∆δ^15^N − 2SD of up to 64.3‰). Nematodes, including Desmoscolecida, were enriched in both diatoms and cyanobacteria treatments, by the greatest amount at 58 days. There was typically only limited or no enrichments in foraminifera with ^13^C or ^15^N after incubation with cyanobacteria or diatoms, even after 58 days (Figure 3).

At 1°N, metazoan meiofauna (i.e., harpacticoid copepods, nematodes, and polychaetes) had modest enrichments with both the phytoplankton types. Some harpacticoids and nematodes were slightly enriched in ^15^N (∆δ^15^N − 2SD of up to 5.1‰; Figure S5; Table S6). However, enrichment in ^13^C was almost negligible (∆δ^13^C − 2SD of at most 0.2‰; Figure 3). Clear enrichment was observed in two samples of foraminifera in Diatom‐51d (∆δ^13^C − 2SD of 59.4‰), but there were no signals of ^13^C enrichment in foraminifera incubated with cyanobacteria.

### 3.3 Prokaryotic and viral responses to the addition of phytoplankton labeled with ^13^C and ^15^N

**FIGURE 4:**
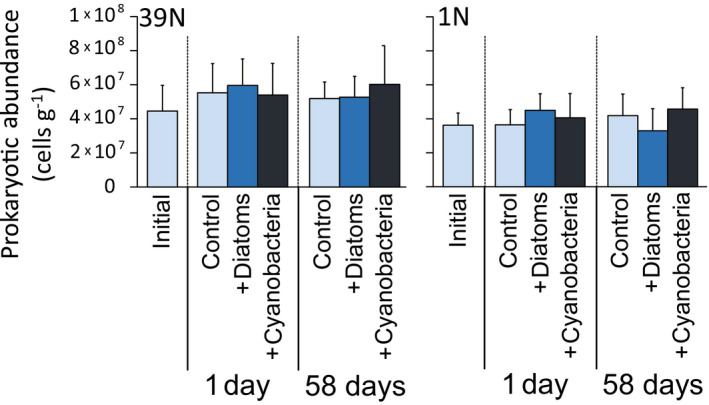
Prokaryotic abundances in surface sediments (top 1 cm) before and after the in situ addition of diatoms or cyanobacteria at 39°N and 1°N at abyssal depths. Reported are the values for the control samples (i.e., background cores retrieved next to the in situ incubation chambers) at the start of the in situ incubation (“initial”) and during the experiments (“control”), as well as the values for the samples obtained from the in situ chambers incubated after the addition of diatoms or cyanobacteria at 39°N and 1°N. Each bar represents the average value (with error bar showing the respective SD) resulting from the analysis of three sediment cores, collected either in one of the available in situ chambers (for the “+Diatoms” and “+Cyanobacteria” treatments) or in background undisturbed sediment spots next to the chambers (for the initial and control sediments). Note that the values of prokaryotic abundance showed no statistical differences between stations, treatments, nor over time (see Table S7)

**FIGURE 5:**
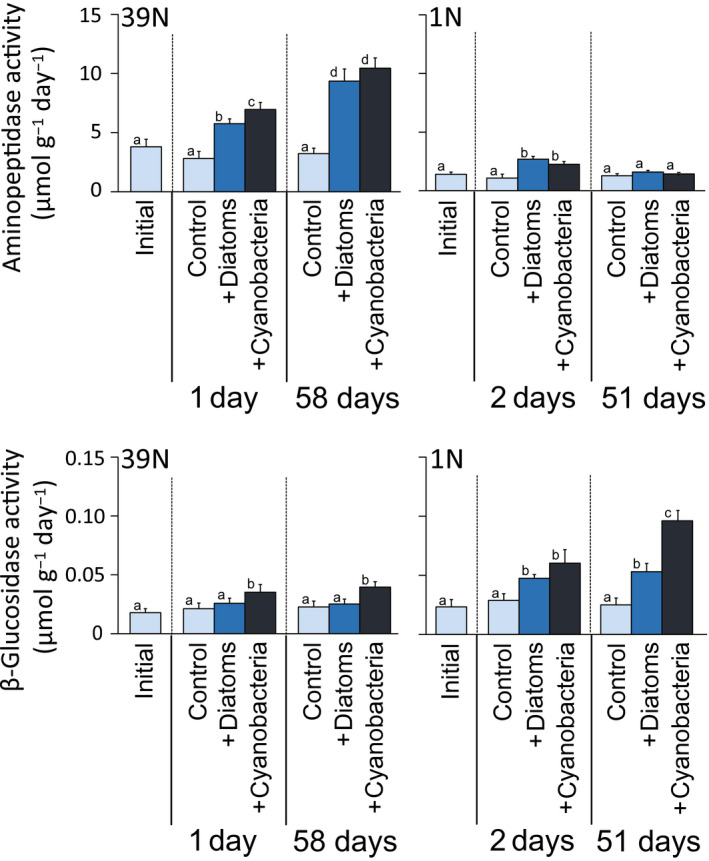
Extracellular enzymatic (aminopeptidase and β‐glucosidase) activities in surface sediments (top 1 cm) as a proxy of the metabolic response of prokaryotes to the in situ addition of diatoms or cyanobacteria, at Stations 39°N and 1°N at abyssal depths. Reported are the values for the control samples (i.e., background cores retrieved next to the in situ incubation chambers) at the start of the in situ incubation (“initial”) and during the experiments (“control”), as well as the values for the samples obtained from the in situ chambers incubated after the addition of diatoms or cyanobacteria at 39°N and 1°N. Each bar represents the average value (with error bar showing the respective SD) resulting from the analysis of three sediment cores, collected either in one of the available in situ chambers (for the “+Diatoms” and “+Cyanobacteria” treatments) or in background undisturbed sediment spots next to the chambers (for the initial and control sediments). The different letters highlight the main statistical differences observed, comparing controls and treatments at each time point and compared with initial values. Note that cyanobacteria addition always increased enzymatic activities up to values similar or higher than diatom addition (see Table S8 for detailed pairwise comparisons)

The abundance of prokaryotes in surface sediment (0–1 cm) did not change significantly after in situ incubations with added diatoms or cyanobacteria, at either 39°N or 1°N (*p* > .05, Figure 4; Table S7). However, both aminopeptidase and β‐glucosidase activities, used as a proxy of metabolic response of prokaryotes (respectively for protein or carbohydrate degradation) to the in situ addition of diatoms or cyanobacteria, increased significantly after the addition of phytoplankton compared with background control samples (Figure 5; Table S8). For aminopeptidase, at 39°N, activity increased significantly in Diatom‐1d (two times compared with control samples) and significantly more (2.5 times) in Cyano‐1d (*p* < .05), and both treatments displayed a further increase at 58 days (to 2.9–3.3 times; Figure 5; Table S8, *p* < .01). At 1°N, aminopeptidase activities showed a significant increase only in the 1 day incubation both for diatom and cyanobacteria addition, up to the same (2.1–2.4 times) increase levels (Figure 5; Table S8). Similar to aminopeptidase, also for β‐glucosidase, we found a higher response to cyanobacterial addition at 39°N, with significant (1.7 times) increase in activity compared with initial and control values (*p* < .05, both at 1 and 58 days), while diatom addition did not result in significant increases (Figure 5; Table S8). At 1°N, β‐glucosidase activity increased (1.7–2.1 times) with the addition of both diatoms and cyanobacteria, but displaying a significantly higher increase (up to 4.2 times) in the longer term after cyanobacterial addition (Figure 5; Table S8).

**FIGURE 6:**
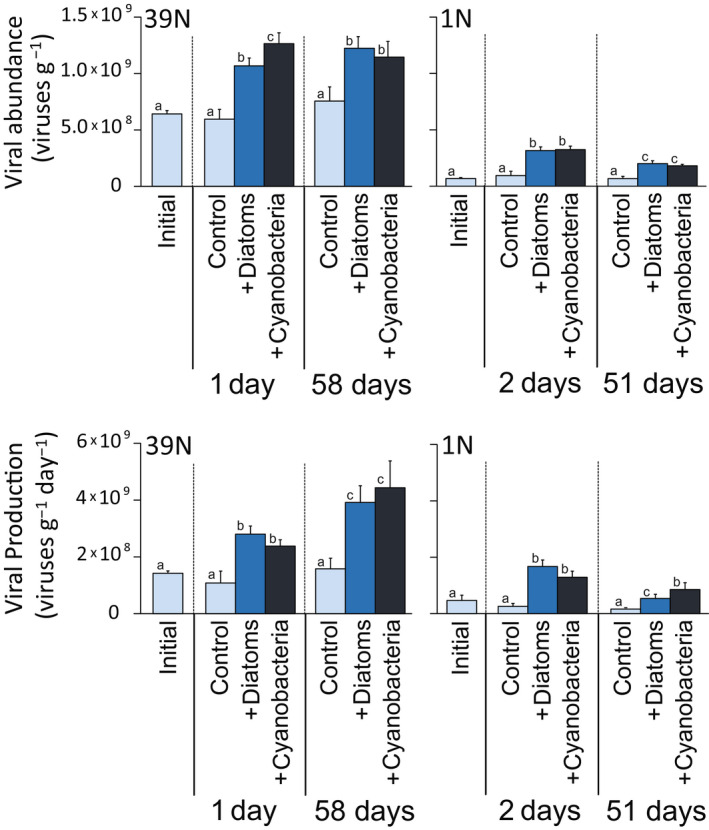
Viral abundance and viral production rates in surface sediments (top 1 cm) before and after the in situ addition of diatoms or cyanobacteria at 39°N and 1°N at abyssal depth. Reported are the values for the control samples (i.e., background cores retrieved next to the in situ incubation chambers) at the start of the in situ incubation (“initial”) and during the experiments (“control”), as well as the values for the samples obtained from the in situ chambers incubated after the addition of diatoms or cyanobacteria at 39°N and 1°N. Each bar represents the average value (with error bar showing the respective SD) resulting from the analysis of three sediment cores, collected either in one of the available in situ chambers (for the “+Diatoms” and “+Cyanobacteria” treatments) or in background undisturbed sediment spots next to the chambers (for the initial and control sediments). The different letters highlight the main statistical differences observed comparing controls and treatments at each time point and compared with initial values. Note that cyanobacteria addition always increased viral abundance and viral production up to values similar or higher than diatom addition (see Table S8 for detailed pairwise comparisons)

As enzymatic activities, also viral abundance and viral production rates increased significantly after the addition of phytoplankton compared with background control samples (Figure 6; Table S8). Viral abundance, at 39°N, increased significantly in Diatom‐1d (1.7 times compared with control samples) and significantly more (2.1 times) in Cyano‐1d, keeping similarly higher values at 58 days (Figure 6; Table S8). At 1°N, viral abundance showed the same increase after the diatom or cyanobacteria addition, with a significantly higher increase at 2d (3.4–3.5 times) than at 51d (2.7–3.0 times; Figure 6; Table S8). At 39°N, viral production rates increased significantly and similarly in the diatom and cyanobacteria addition (2.2–2.6 times at 1d and significantly more at 58d, with a maximum of 4.9 times for the cyanobacteria treatment; Figure 6; Table S8). At 1°N, viral production rates increased similarly in the diatom and in the cyanobacteria treatment at 2d (5.1–6.6 times), while the cyanobacteria treatment maintained a higher increase compared to that of the diatom treatment (5.3 vs. 3.3 times) at 51d (Figure 6; Table S8).

## 4 DISCUSSION

**FIGURE 7:**
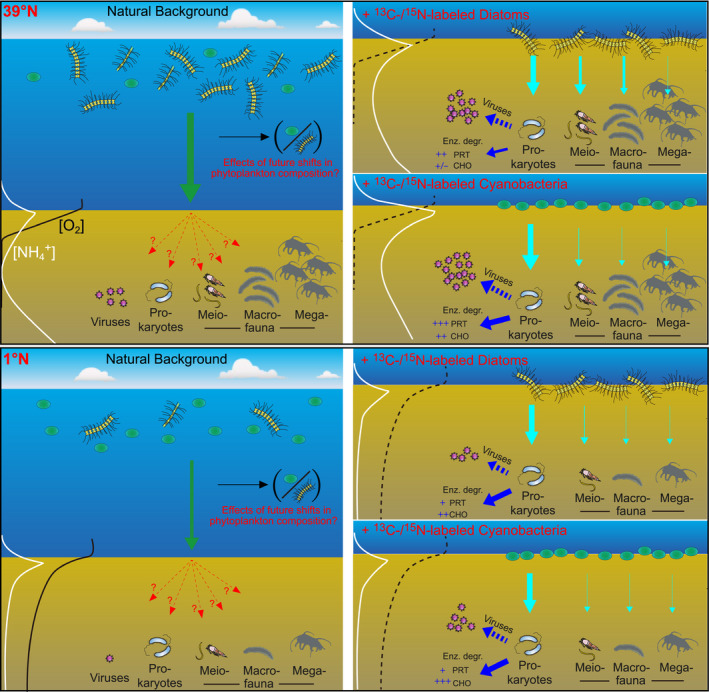
Schematic view of the natural (background) settings of both 39°N and 1°N sites, as well as a synoptic view of the main highlights from the in situ incubation experiments conducted in this study on the responses of abyssal benthic biota to shifts in phytodetritus compositions linked to global climate change. The scheme summarizes results reported in detail in previous sections of this manuscript. Bigger arrows indicate a larger/increased role in the processing of labeled phytoplankton. Enz. degr.: Enzymatic degradation of phytodetritus either by aminopeptidase activity (PRT) or by β‐glucosidase activity (CHO). Dotted arrows from prokaryotes to virus indicate viral production. As reported in the studies by Amaro et al. (2019) and Nomaki et al. (2021) from the comparison among values obtained from background sediments samples collected at 39°N and 1°N, megafaunal abundance was roughly 36 times higher at 39°N, macrofaunal abundance and biomass were 5.4 and 25 times, respectively, higher at 39°N, meiofaunal abundance and biomass were 3.5 times and 1.6 times, respectively, higher at 39°N, prokaryotic abundance was comparable between 39°N and 1°N, and viral abundance was approximately 10 times higher at 39°N

Our experiments conducted in situ at the abyssal seafloor revealed that the currently projected shifts in phytoplankton compositions linked to global climate change (and especially the relative increase in picophytoplankton in surface oceans expected at higher latitudes) have the potential to profoundly alter the current functioning of abyssal benthic ecosystems. A graphic scheme is provided in Figure 7, with a synoptic view of the main results obtained in this study. In the following discussion paragraphs, we detail the many novel insights that were obtained from our multifactorial experiment conducted in situ at abyssal depth, including the responses of abyssal biota from viruses to megafauna to different types of phytoplankton supply in either eutrophic or oligotrophic oceanographic sectors, at different timescales.

Our results show that the different components of the benthic abyssal biota displayed different responses to the addition of diatoms or cyanobacteria as analogous of phytodetritus. While megafauna played overall a minor role in phytodetritus consumption at both Stations 1°N and 39°N, macro‐ and meiofauna generally contributed more, with a larger consumption of phytodetritus at 39°N than at 1°N, and showing evident preference for diatoms over cyanobacteria (Figures 2, 3, and 7). Although similar direct evidence on food source preferences for abyssal organisms is largely lacking to date (e.g., Enge et al., 2011; Witte et al., 2003), some benthic foraminifera are known to feed selectively upon diatoms (Austin et al., 2005; Nomaki et al., 2006), suggesting that diatoms may be a preferred energy and carbon source for foraminifera and possibly other abyssal faunal taxa.

Contrarily to the response of abyssal eukaryotes, we show that prokaryotes and viruses generally displayed similar or even stronger responses to cyanobacterial than to diatom supply. Indeed, the addition of cyanobacteria rather than diatoms stimulated prokaryotic β‐glucosidase activities at both 1°N and 39°N sites, also triggering the greatest increases in aminopeptidase activities at 39°N (Figures 5 and 7). Similarly, viral abundance and production rates increased after cyanobacteria additions at both sites, at similar or higher values compared to diatoms additions (Figures 6 and 7). The ∆δ^13^C values of DIC at 39°N indicated that there had been substantial mineralization of isotopically labeled cyanobacteria within 1 day after the cyanobacteria input (Figure S4), while none of the benthic eukaryotes investigated ingested substantial amounts of isotopically labeled cyanobacteria after 1 day. We thus conclude that the uptake of cyanobacteria and subsequent mineralization by prokaryotes was mostly responsible for the observed mineralization of OC. These results suggest that, considering current climate change scenarios leading to an increasing relative importance of picophytoplankton (e.g., cyanobacteria) as main primary producers in surface oceans, benthic deep‐sea eukaryotes could be largely affected by the predicted relative decrease in diatoms especially at higher latitudes. A decrease in their favorite food source can lead to decrease in eukaryotic activity and abundance, exacerbating food limitation in deep‐sea sediments (Danovaro, Corinaldesi, Dell'Anno, & Rastelli, 2017; Gooday & Jorissen, 2012). These changes could alter the composition and functions of the entire food web, as benthic small‐sized eukaryotes are important trophic links between phytodetritus and higher trophic levels in the oceans (Gudmundsson et al., 2000; Herbert, 1991; Nomaki et al., 2021).

At the same time, our results show that prokaryotes and viruses will be unaffected by an increase in cyanobacterial supply, thus increasing their quantitative role and organic carbon cycling rates. In turn, such a projected increase in the OC processed through the benthic microbial loop and the hampering of its transfer up the food chain to metazoans will likely have negative effects on the oceanic food web functioning, as well as their biodiversity and overall carbon sequestration capacity (Danovaro, Corinaldesi, Dell'Anno, & Rastelli, 2017). Even if our results point out that the projected changes in the composition of phytodetritus composition will especially affect the benthic abyssal biota at higher latitudes, we can expect that the alteration in the functioning of large oceanic sectors will have overall effects at the global scale.

Notably, the risk of food limitation caused by the expected shifts in phytodetritus composition will add to the projected decrease in POC fluxes and, consequently, the overall quantity of OM reaching the seafloor (Buesseler et al., 2007; Moran et al., 2010, 2015; Steinacher et al., 2010). Further studies are needed to assess the synergistic effects of the concurrent 32% decline in POC fluxes predicted for the Pacific abyssal seafloor by 2100 (Sweetman et al., 2017), and of other climate‐induced changes on the functioning of pelagic ecosystems (Buesseler et al., 2007; Lefort et al., 2015; Moran et al., 2010, 2015; Steinacher et al., 2010).

Declines in the activity and abundance of eukaryotes, particularly of the benthic macrofauna, also imply a possible decrease in sediment mixing (Arndt et al., 2013; Middelburg, 2018). The results obtained in this study from the tracing of the labeled phytodetritus, suggest that benthic fauna such as annelids that are relatively abundant at the investigated sites (Nomaki et al., 2021), played a relevant role in mixing of phytodetritus along the vertical sediment profile (Levin et al., 1997). Future investigations should better elucidate how changes in food supply could affect bioturbation and alter the use of phytodetritus and OC burial in abyssal sediments (Arndt et al., 2013; Middelburg, 2018).

A second major result highlighted by our study is that prokaryotes and viruses responded very rapidly (e.g., within 1–2 days) to phytoplankton supply, in terms of changes in their abundance and activities (Figures 5 and 6). Similarly, the uptake of isotopically labeled diatom and cyanobacteria was generally higher in smaller organisms like meiofauna, relative to macro‐ and megafauna (Figures 2 and 3). Even though differences in body sizes may contribute to explain this result (as larger organisms that incorporate isotopically labeled food in same amounts as smaller organisms should show relatively lower ^13^C or ^15^N concentrations in their biomass), previous studies reported that significant enrichments in macrofauna (e.g., Levin et al., 1999; Woulds et al., 2009) relative to smaller organisms. This suggests that the low enrichments in ^13^C or ^15^N of macrofauna found in this study were not explained by such a “dilution effect” alone. Similarly, at both latitudes, we found rapid responses of the microbial components (including prokaryotes and viruses) to diatom and cyanobacteria input, in terms of changes in their numbers or activities (Figure 5). Notably, at the Porcupine Abyssal Plain in the North Atlantic Ocean, Witte et al. (2003) reported that the addition of labeled phytoplankton did not cause a rapid (within 2.5 days) response by bacteria (used by the authors as synonym of heterotrophic prokaryotes) in either β‐glucosidase activities or ^13^C assimilation into bacterial fatty acids. Previous findings thus differ from our results, which conversely indicate a fast and significant increase in prokaryotic enzymatic activities within 1–2 days at both 1°N and 39°N (Figure 5). The rapid increase in prokaryotic enzymatic activities after the addition of phytoplankton in this study is consistent with previous reports of microbial responses to phytodetritus deposition (Beaulieu, 2002; Gooday & Turley, 1990). The differences in the responses of the prokaryotes to the labeled diatoms between the North Atlantic and the Western Pacific could be explained by the difference of the benthic assemblages in these two areas, as well as to the potential effects of the different phytoplankton material added (as Witte et al. added a suspension of freeze‐dried *Thalassiosira* diatoms, whereas we added frozen and thawed *Chaetoceros* diatoms or cyanobacteria). Moreover, as phytodetritus can also vary in terms of its size and organic composition (Beaulieu, 2002), future studies should consider testing not only different phytoplankton taxa but also different sizes and quality of the added phytoplankton.

Despite, in our study, phytoplankton input at the abyssal seafloor enhanced the benthic prokaryotic metabolism as suggested by the increase in the rates of enzymatic activities (Figure 5) and biomass turnover, prokaryotic abundance remained rather constant (Figure 4). This discrepancy could be explained by the increased rates of viral production and prokaryotic mortality (Corinaldesi et al., 2012; Danovaro et al., 2008), which counterbalanced the positive effect of the phytoplankton supply on prokaryotic metabolism and biomass turnover.

Smaller organisms tend to have shorter generation times, thus their net contribution to C processing can be higher than expected from the mere changes in their abundance or biomass. In contrast, larger organisms generally have slower metabolic rates and longer turnover times and reproductive rates, thus resulting in amounts of POC metabolized per unit biomass greater for prokaryotes than for larger organisms (Gooday et al., 2008; McClain et al., 2012). These results are consistent with previous evidence of similar prokaryotic abundances at 39°N, 1°N, and 12°N in the Pacific Ocean, despite the higher POC fluxes to the seafloor and higher prokaryotic and viral activities at the eutrophic 39°N compared to the other two oligotrophic sites (Nomaki et al., 2021).

A secondary, though relevant, pattern highlighted by our results is that, at the eutrophic area 39°N, aminopeptidase activities were largely increased by the phytoplankton additions, while displaying a much lower response at the 1°N oligotrophic area (Figure 5; Table S8). Conversely, β‐glucosidase activities exhibited larger increases to the phytoplankton inputs at 1°N than at 39°N. Nomaki et al. (2021) reported that aminopeptidase activities of natural background abyssal sediments showed highest values at the eutrophic site (39°N) and lowest values at an ultra‐oligotrophic site (12°N), while the opposite was observed for β‐glucosidase activities. Such evidence suggests that the observed differences in the metabolic potential of the benthic prokaryotic assemblages at the sites investigated reflect the differences in the surface ocean productivity between eutrophic and oligotrophic areas, as well as the differences in OM quantity and quality at the respective abyssal seafloor sites (Amaro et al., 2019; Nomaki et al., 2021). In particular, we can hypothesize that, at the eutrophic site 39°N, the observed higher relevance of proteins (both as quantity and relative fraction of biopolymeric C; Amaro et al., 2019) might favor protein‐degrading prokaryotes and related catabolic processes based on aminopeptidase. Conversely, the lower concentrations of proteins and the higher relative fraction of carbohydrates at the oligotrophic site (Amaro et al., 2019), likely including particularly refractory polysaccharides (Hedges et al., 2001), could select for prokaryotes especially adapted to glucosidase‐based catabolic processes. Further studies coupling stable isotope‐probing techniques with next‐generation DNA/RNA sequencing are needed to deepen our knowledge on the response of the benthic prokaryotes in terms of assemblage composition and metabolism under different trophic constraints and changes in the composition of downward POC fluxes due to global climate change.

Overall, our study provides the first in situ information on the possible effects of shifts in phytoplankton (and consequently, phytodetritus) composition linked to global climate change. Based on the in situ experimental studies at different oceanographic sectors with different timescales, we showed that different components of the benthic abyssal biota can have different responses. Our results suggest that a future increase in the relative contribution of picophytoplankton to the POC fluxes reaching the benthic abyssal ecosystems will stimulate prokaryotes and viruses, while negatively affecting eukaryotes such as meiofauna and macrofauna. That is, the smaller the size of the phytoplankton, the more advantaged the microbial components (Moran et al., 2015). These findings suggest that larger size organisms will be the most impacted group by the shifts in food supply linked to global climate change and monitoring them over time series at abyssal depths could provide crucial insights into the effects of climate change on deep‐sea ecosystems (Danovaro et al., 2020; Gambi et al., 2017). At the same time, the relative increase in the importance of benthic prokaryotes and viruses in the processing of phytodetritus at abyssal plains will likely contribute to hamper OC transfer up the food chain to metazoans, further exacerbating the prospected negative effects on the functioning of oceanic food webs, their biodiversity and overall carbon sequestration capacity.

## 5 SUMMARY

At two abyssal stations in the Western Pacific Ocean, we investigated the responses of the benthic biota, from viruses to megafauna, to the input of isotopically labeled diatoms or cyanobacteria, used as analogues of key food sources for deep‐sea biota. At both sites, irrespective of the type of phytoplankton addition, prokaryotic metabolism responded positively and rapidly, playing a relevant role in determining the fate of the phytodetritus supply at the abyssal seafloor. Similarly, viruses increased their replication rates and abundances. At 39°N, the relatively eutrophic site, meiofauna and macrofauna assemblages assimilated both diatoms and cyanobacteria, but with a preference for diatoms. At 1°N, the relatively oligotrophic site, only some of the meiofaunal taxa assimilated cyanobacteria, whereas macrofauna and megafauna assimilated no significant amounts of either diatoms or cyanobacteria. Overall, while many eukaryotes preferred diatoms, prokaryotes preferred cyanobacteria. These findings suggest that a shift in phytodetritus composition from diatoms to cyanobacteria could have negative impacts on the abundance and activities of benthic eukaryotes, especially at higher latitudes, while enhancing prokaryotic activities and OM cycling. Overall, we conclude that such changes have the potential to significantly alter the marine food webs and affect carbon sequestration capacity of the oceans, their biodiversity and ecosystem functioning at the global scale.

## CONFLICT OF INTEREST

The authors declare there is no conflict of interest for this article.

## Supporting information

Fig S1‐5

Table S1‐9

Supplementary Material

## Data Availability

The data that supports the findings of this study are available in the [Link], [Link], [Link] of this article.
