# Supplementary figures and images for: In situ experimental evidences for responses of abyssal benthic biota to shifts in phytodetritus compositions linked to global climate change
Source: Glob Chang Biol. 2021 Sep 23;27(23):6139–55. doi: 10.1111/gcb.15882 (PMC9293103; doi:10.1111/gcb.15882)

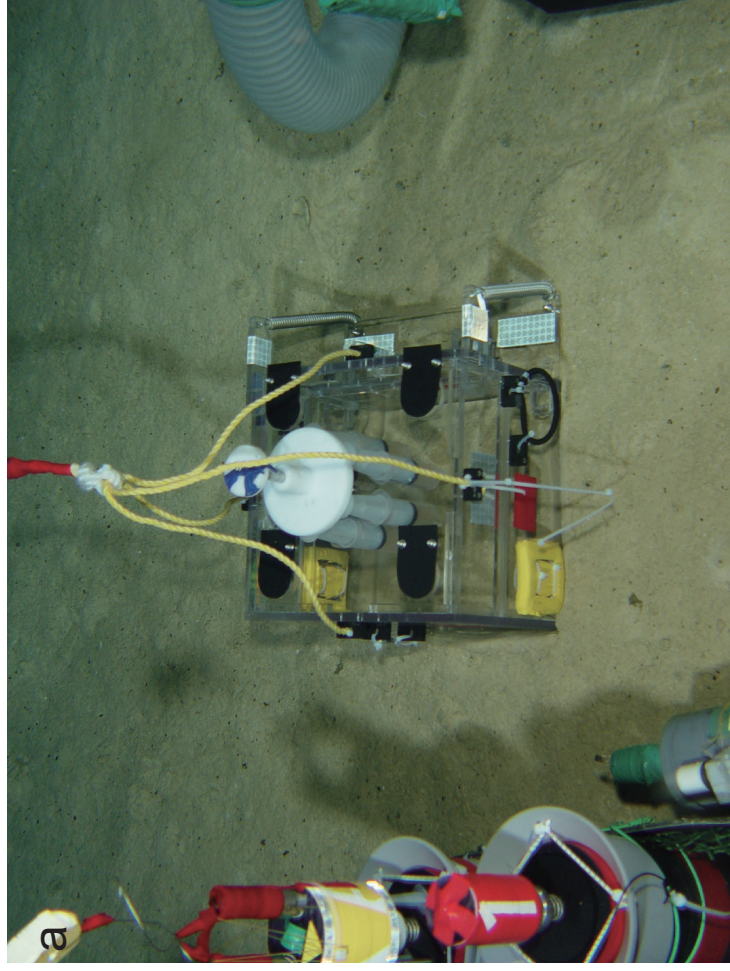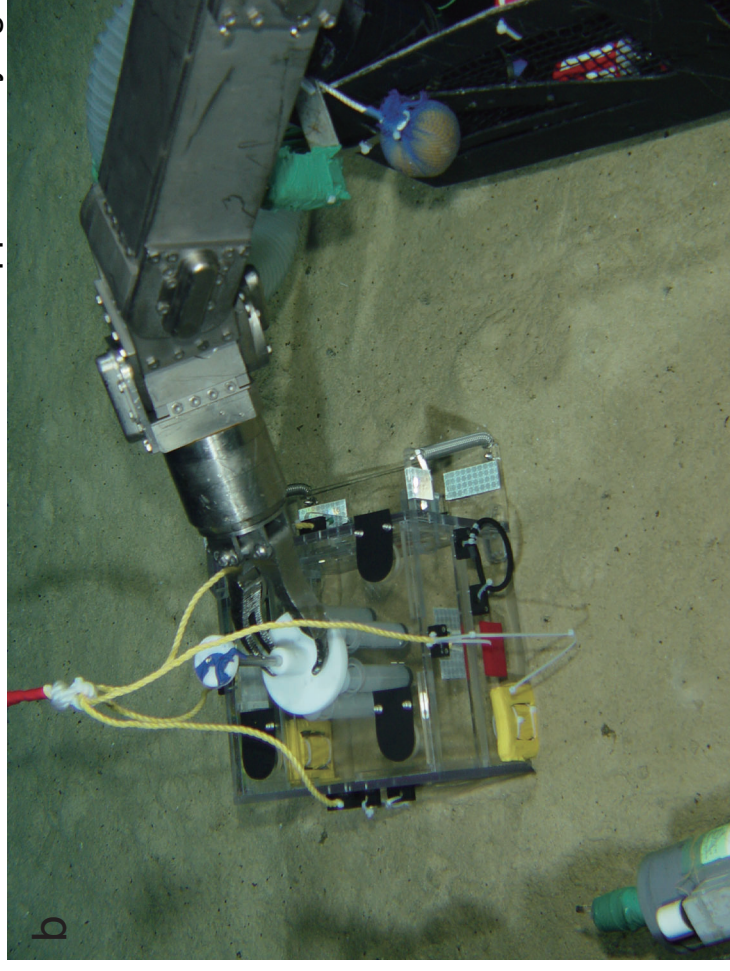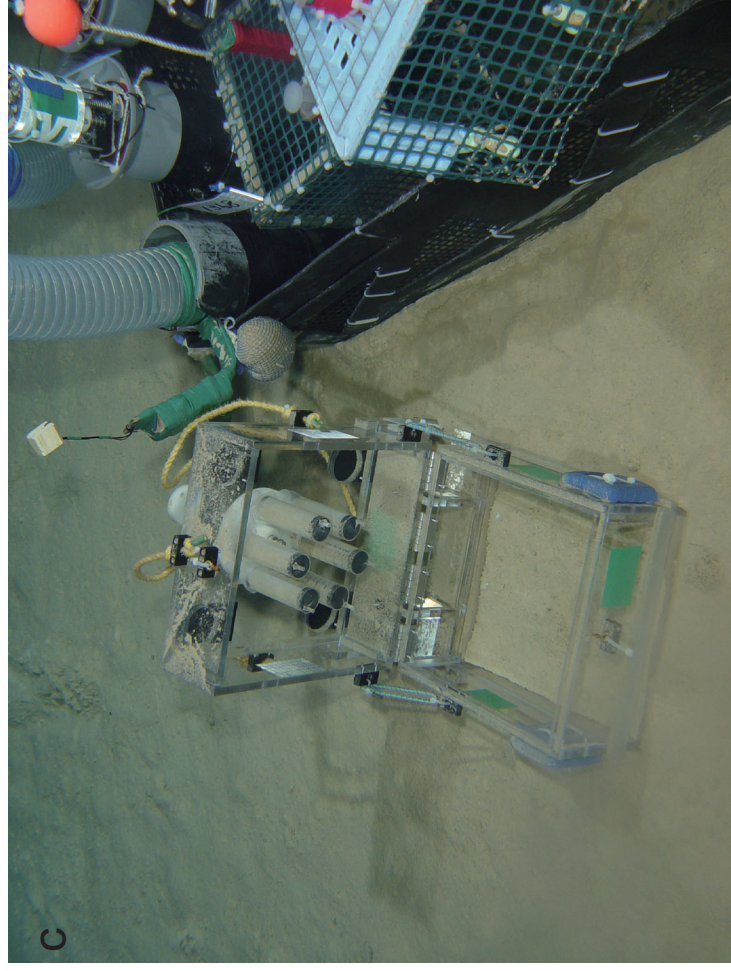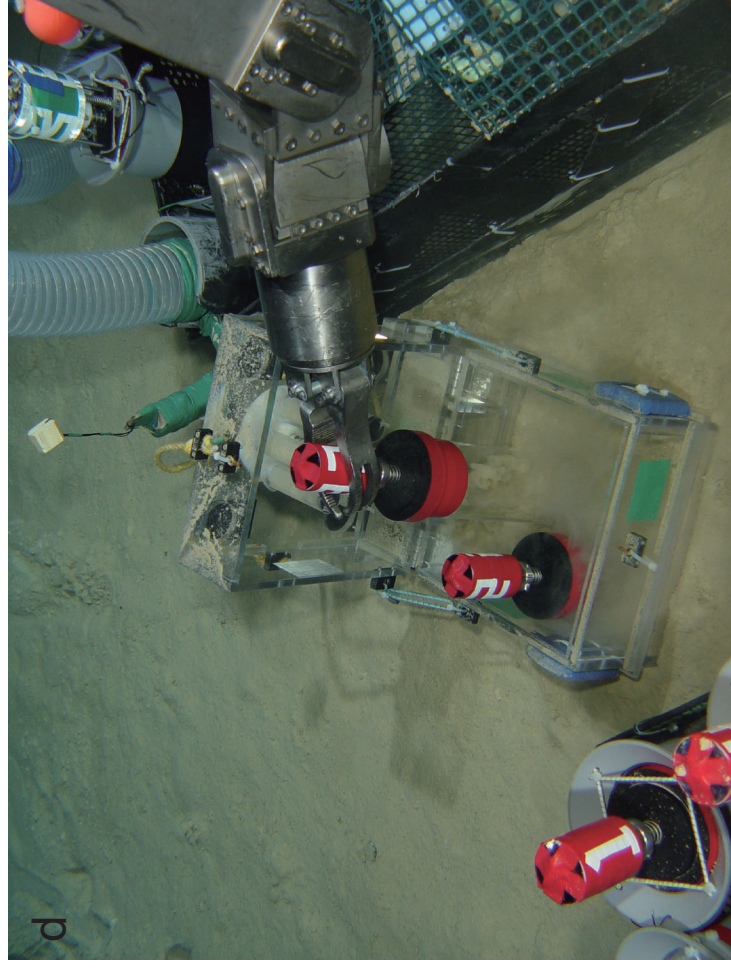

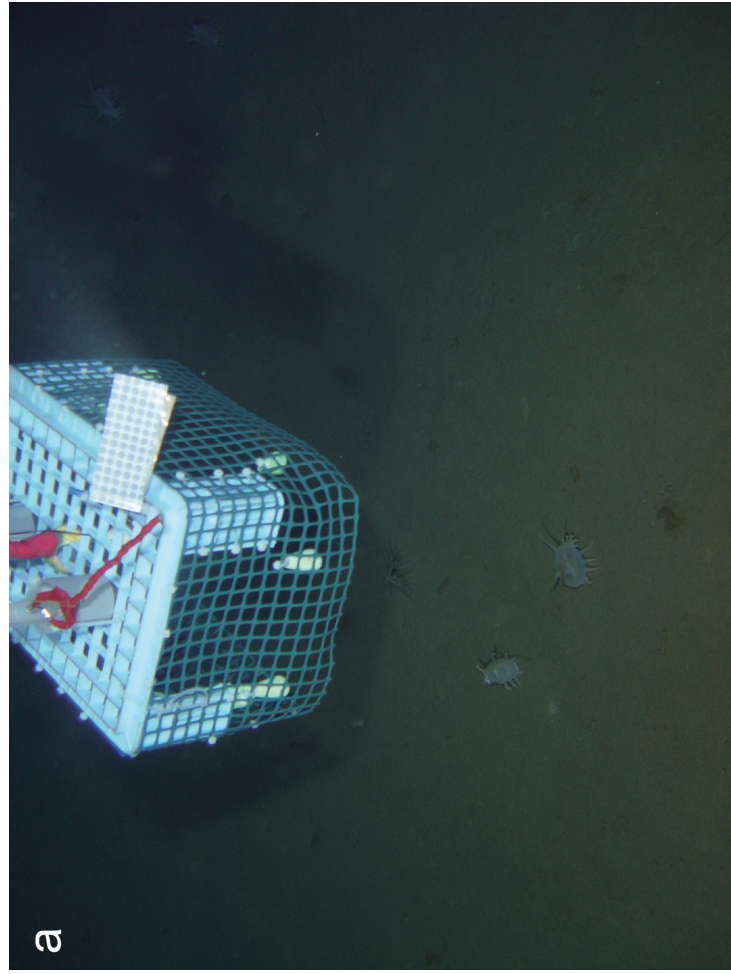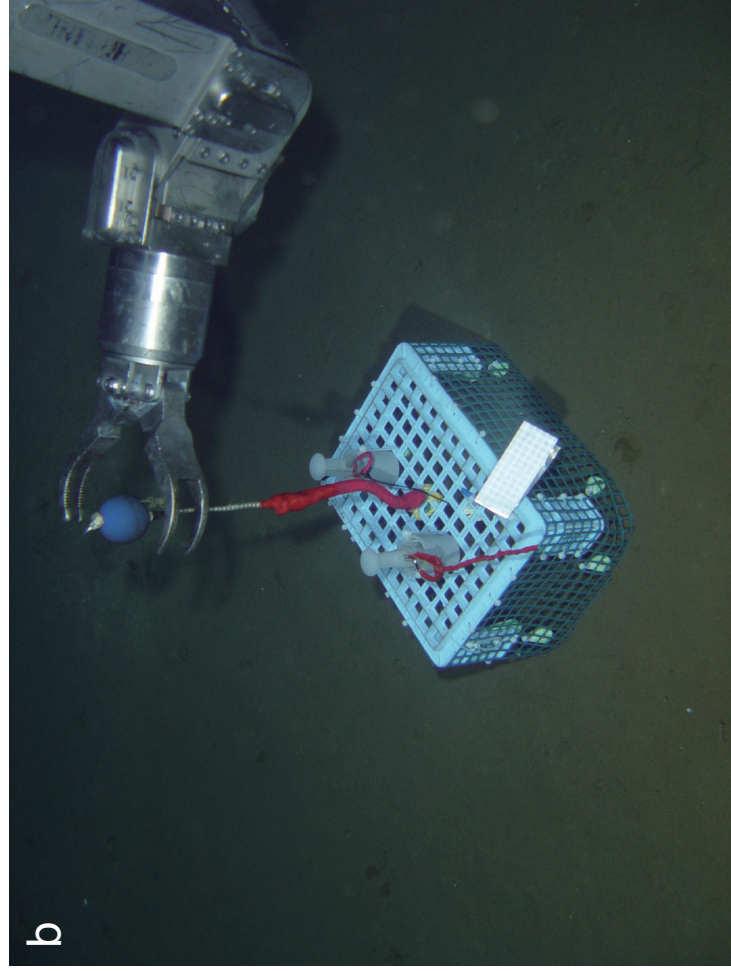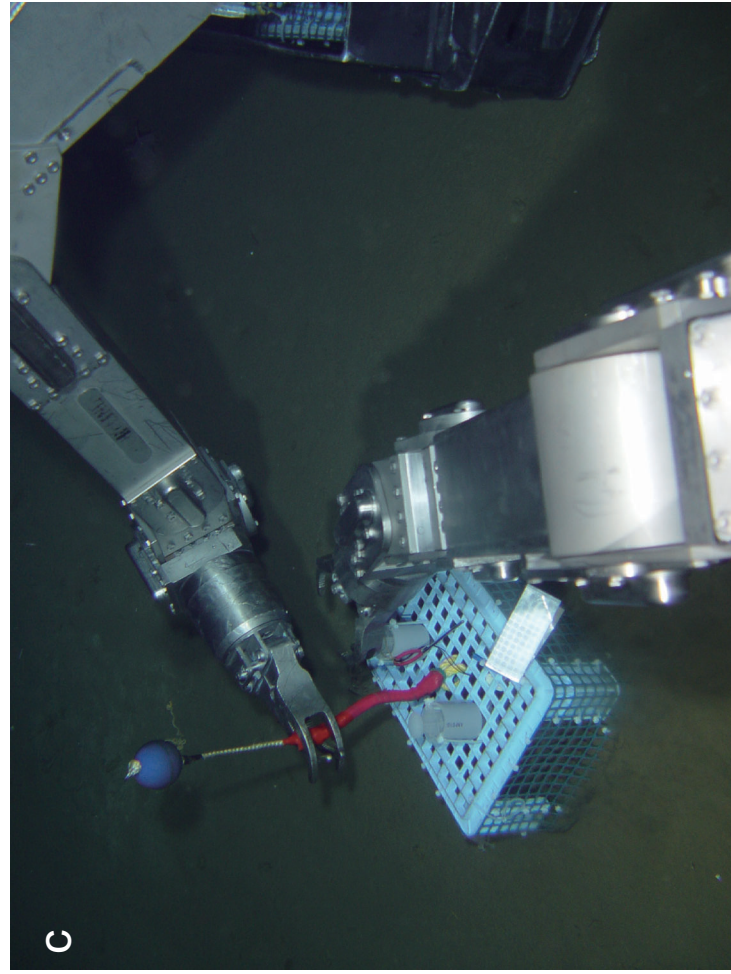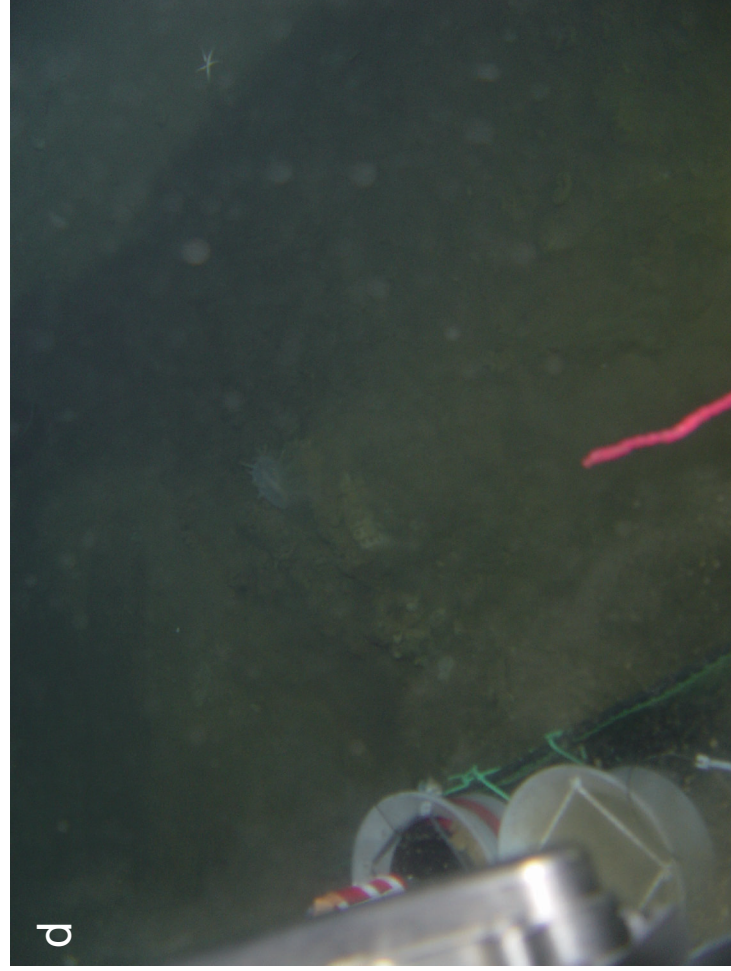

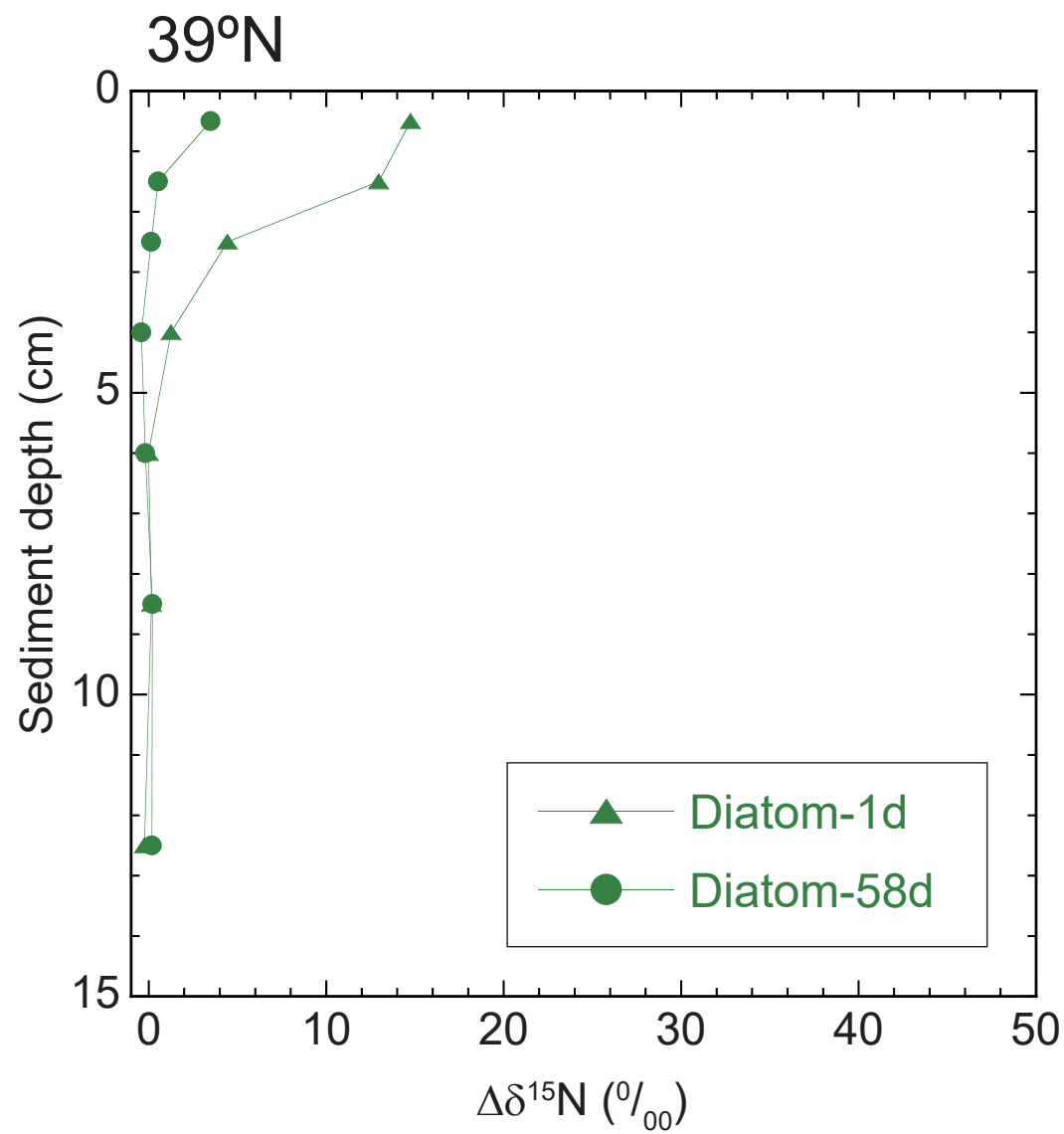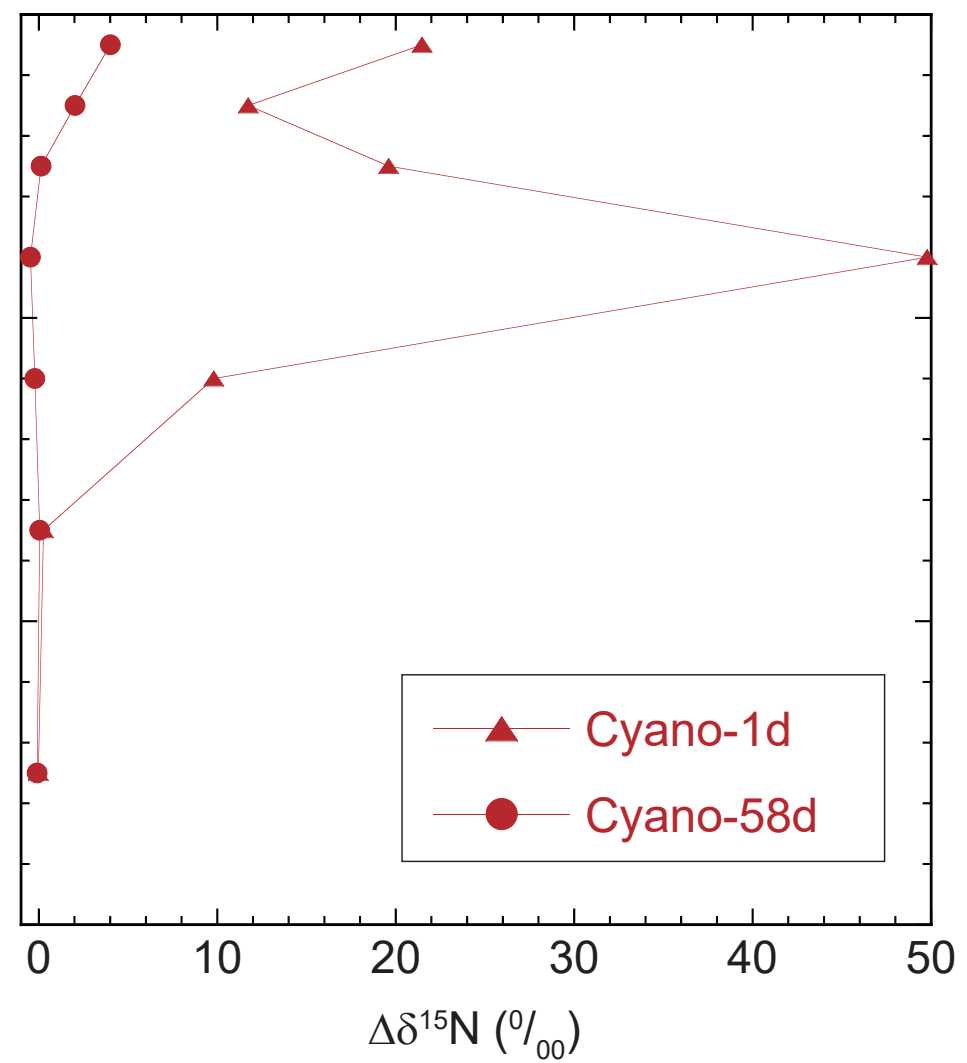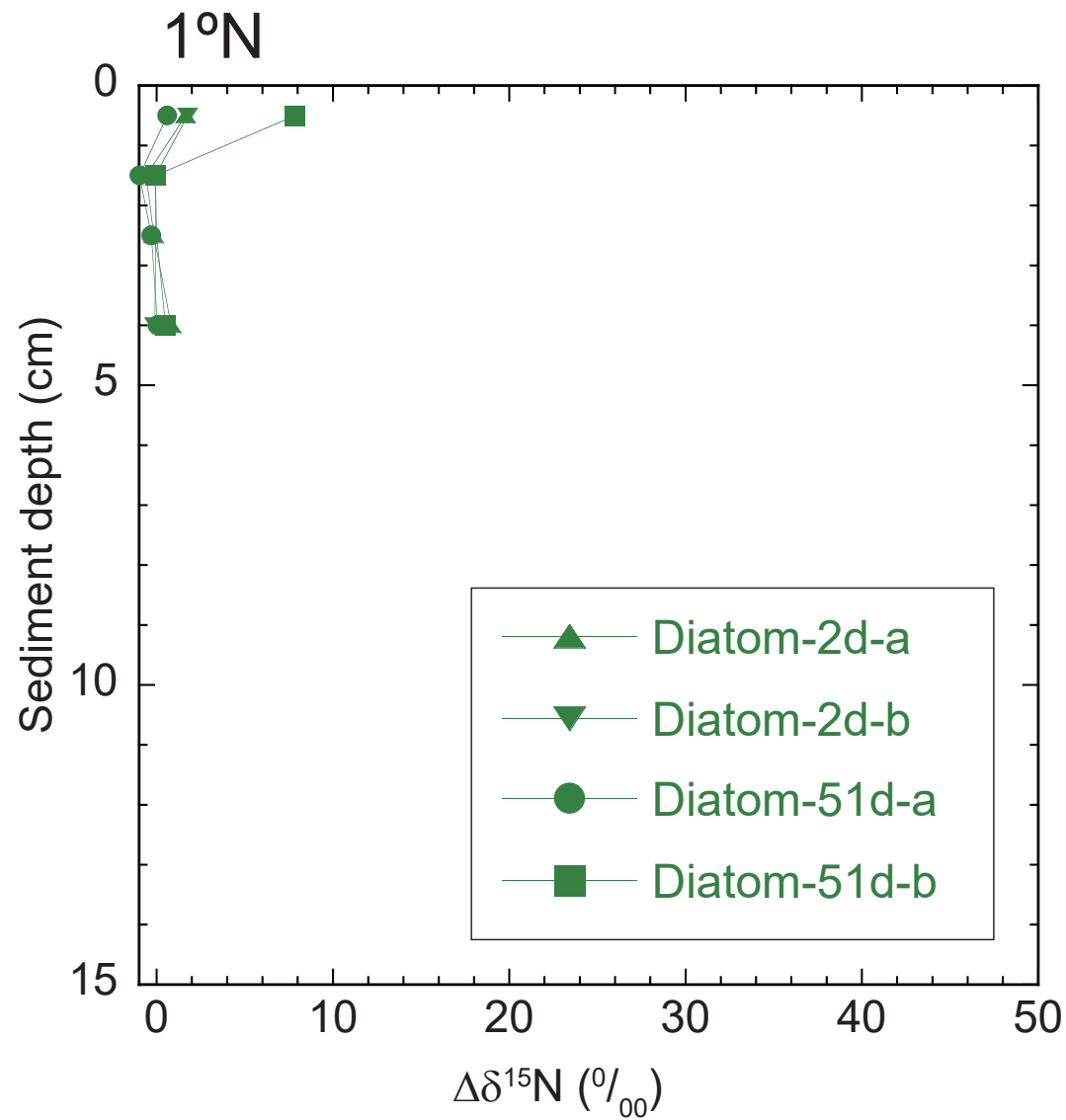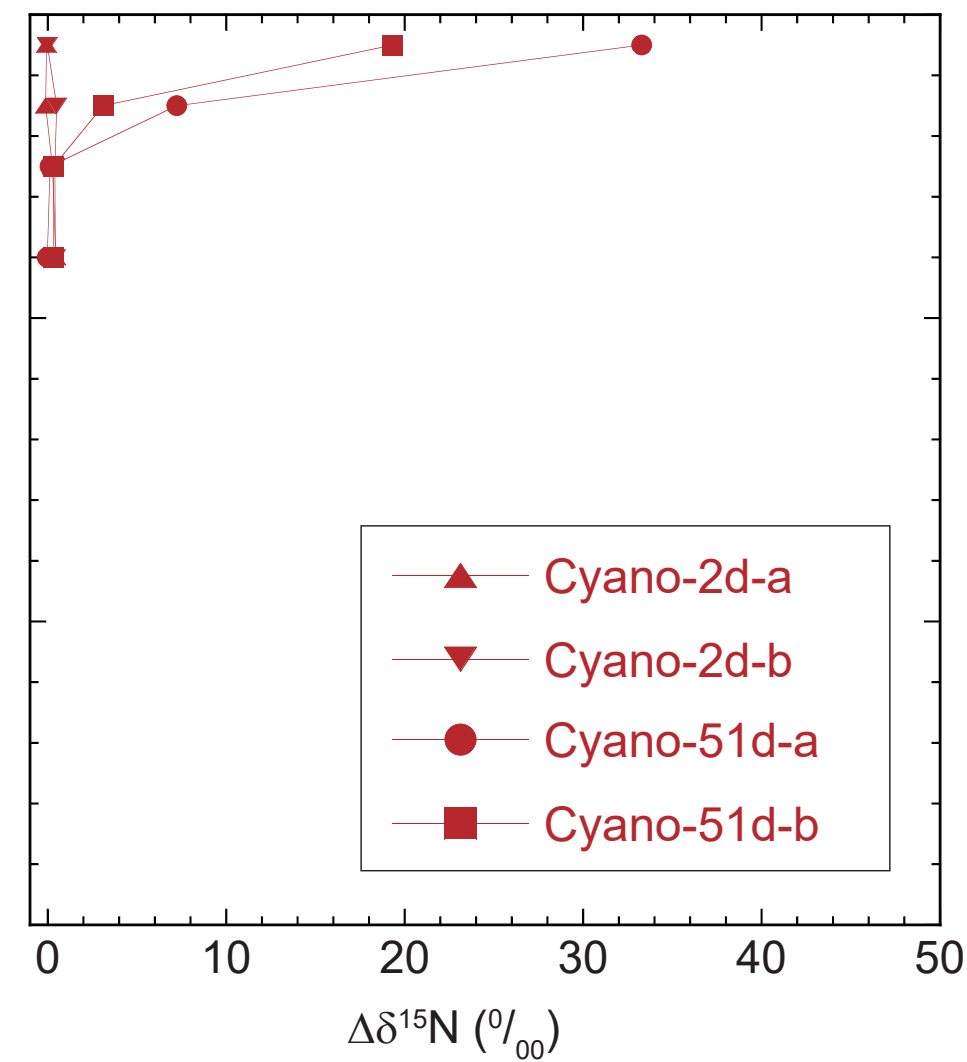

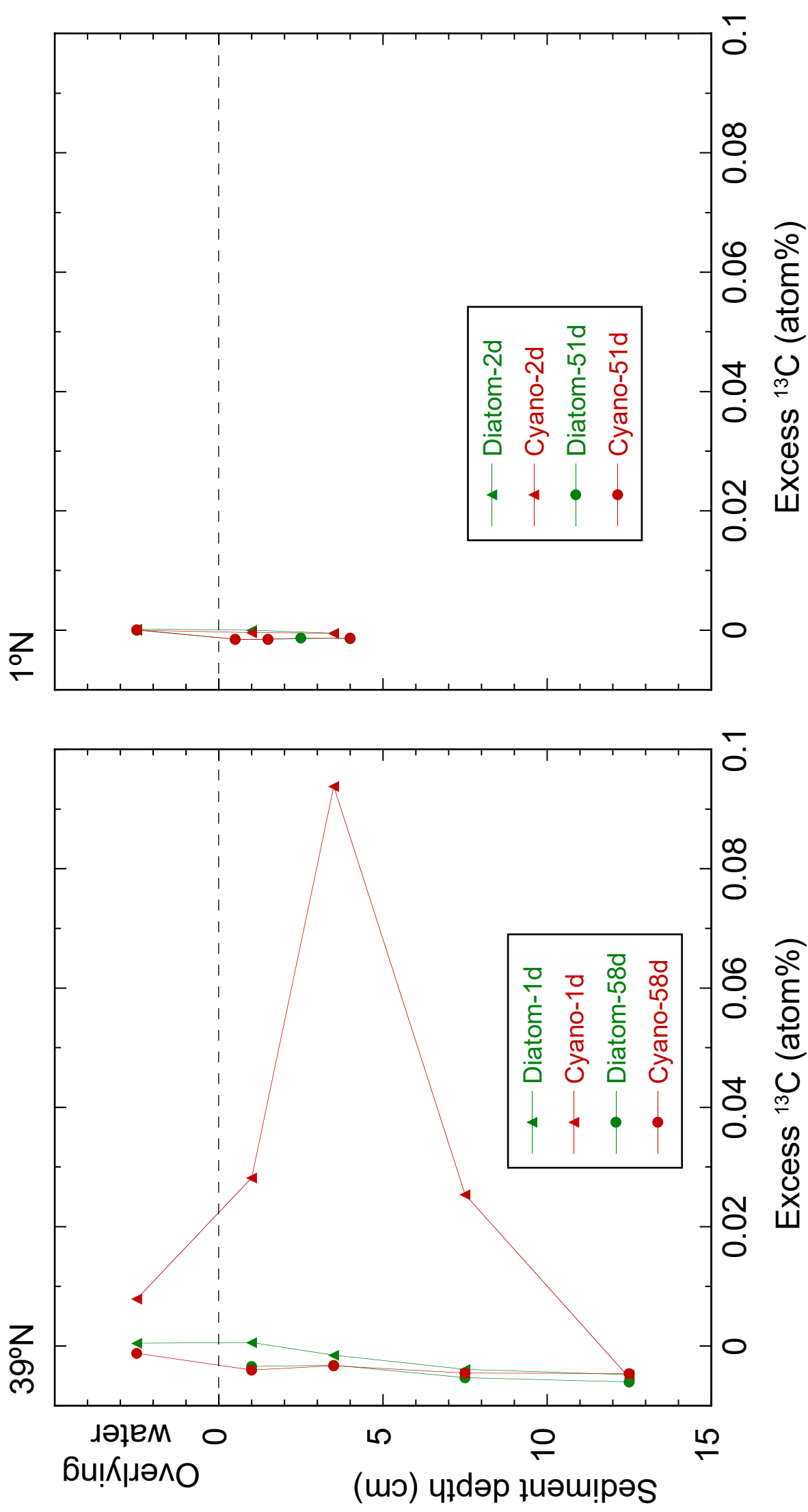

39°N

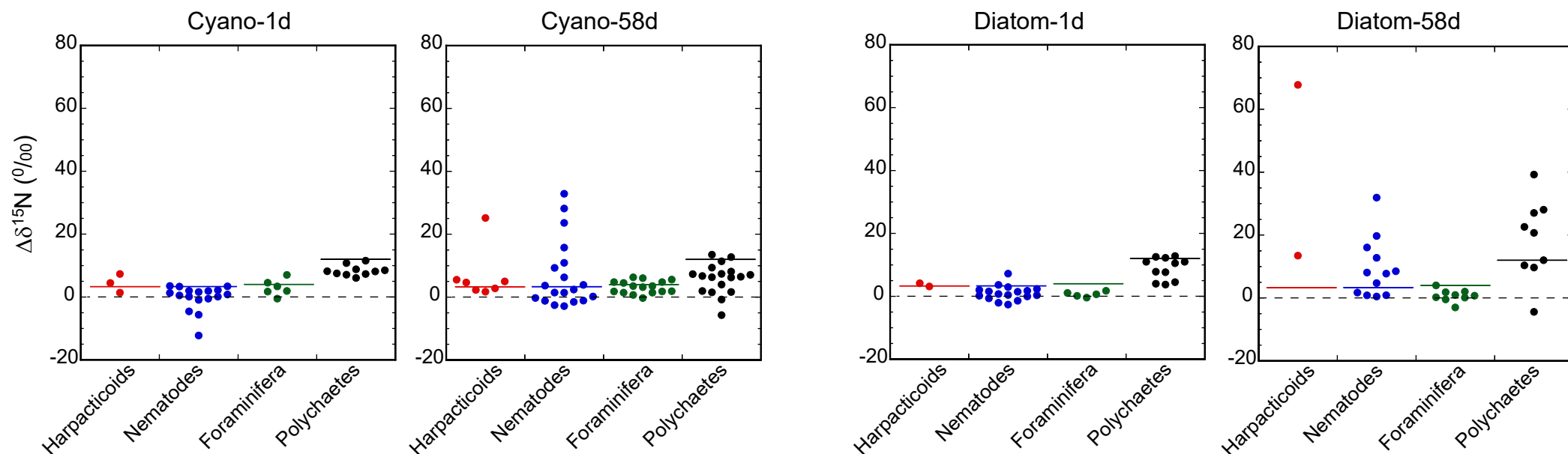

1°N

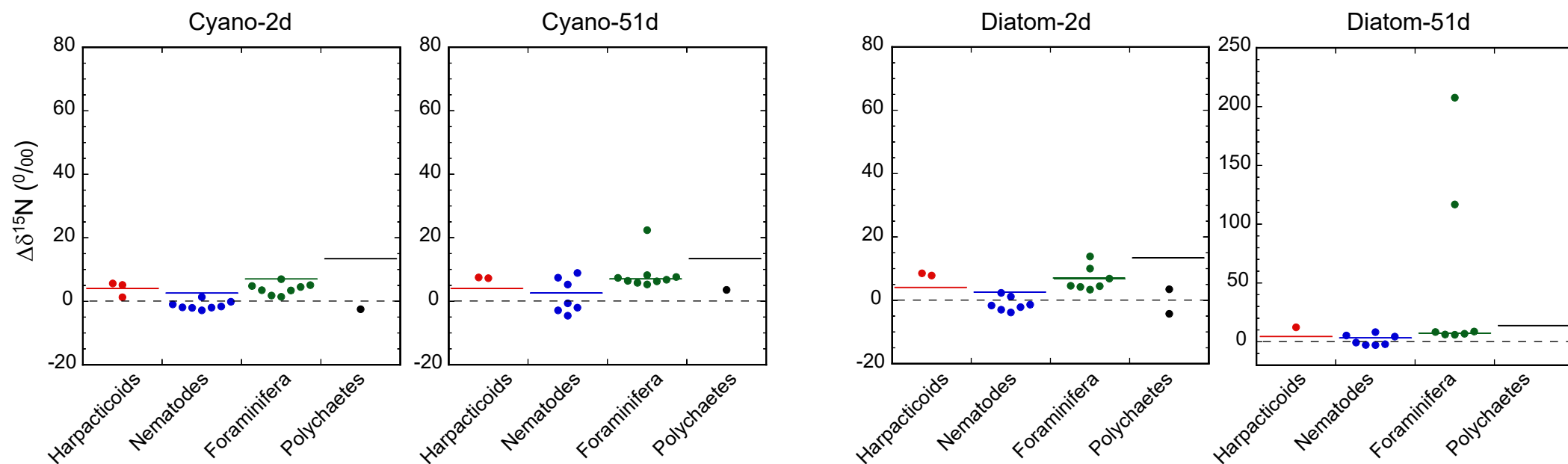

Supplement: Supplementary file 1 — Fig S1‐5 [file GCB-27-6139-s001.pdf]
