# Supplementary material for: In situ experimental evidences for responses of abyssal benthic biota to shifts in phytodetritus compositions linked to global climate change
Source: Glob Chang Biol. 2021 Sep 23;27(23):6139–55. doi: 10.1111/gcb.15882 (PMC9293103; doi:10.1111/gcb.15882)
Supplement: Supplementary file 3 — Supplementary Material [file GCB-27-6139-s003.docx]

SUPPLEMENTARY FIGURE 1. Images of the open-box-type incubation chamber and its deployment on the seafloor. (A) Deployment on the seafloor. (B) Injection of phytoplankton from the syringe. (C) Lid opened automatically 1 day after deployment by a galvanic-timed release mechanism. (D) Sediment sampling using push cores.

SUPPLEMENTARY FIGURE 2. Images of the mesh-type incubation chamber for holothurians and its deployment on the seafloor. (A) Holothurians before chamber deployment (the individual on the right side was placed in the chamber). (B) Deployment of the chamber on the holothurian. (C) Injection of phytoplankton from the syringes. (D) A holothurian below the chamber.

SUPPLEMENTARY FIGURE 3. Profiles of ∆δ^13^C and ∆δ^15^N (differences in values between experimental samples and natural background samples) of bulk sediments at stations 39°N and 1°N.

SUPPLEMENTARY FIGURE 4. Profiles of Excess ^13^C atom% of dissolved inorganic carbon in the pore water extracted from sediments after isotopically labeled phytoplankton were added at stations 39°N and 1°N. Value above 0 cm indicates excess ^13^C atom% of the water overlying the cores.

SUPPLEMENTARY FIGURE 5. Enrichments of ^15^N in meiofauna after 1 and 58 days (39°N) or 2 and 51 days (1°N) following phytoplankton additions. The colored bars indicate ∆δ^13^C values greater than the average + 2σ (SD) of natural background samples (Nomaki et al. 2021).
